# National University

**Published:** 1871-04

**Authors:** 


### NATIONAL UNIVERSITY.

We learn from our exchanges that there is a scheme for the inauguration of a National University, with a medical department, to which all persons, irrespective of color and sex are to be admitted, free of charge. We are the advocates of education. An educated people can alone illustrate the principles and perpetuate the institutions of civil and religious liberty. But while we deplore ignorance, as the twin sister of vice, yet new schemes weighted with crude conceptions and burdened by fanaticism, cannot meet the demands of either civilization or religion. With the scheme of a National University outside of its medical department, we leave for others to discuss. We propose to notice this scheme only so far as it relates to medical education, in its invasion, as we think, of the rights, interests and prerogatives of a profession, which is now, by voluntary association, controlled by laws designed for its advancement and protection. While we do not object to States extending aid to institutions of learning, and while we think they have been remiss in furnishing adequate assistance to medical institutions, we nevertheless affirm that medical law demands that the profession shall accept of such aid only upon those terms which its law shall recognize. This is one of the great distinctions between the regular and irregular institutions. Regular institutions conform to medical law. Before the law of the land all chartered institutions are legal. The corporators must conduct them strictly according to the legal requirements of their respective charters. But the simple fact of an institution being legal, does not make it professional. Eclectic or homœpathic institutions are legally authorized to teach the dogmas peculiar to their several systems, but they are never recognized, nor never can be, as regular—they are *regular* before the law, but irregular before the profession. Should the Legislature of any State grant a charter to any institution, which would confer the legal right to such institution to blend together all (so-called) systems of medicine: To have professorships for teaching allopathy, (?) hydropathy, tompsonism homœpathy, and the "laying on of hands," such charter would be legal. Not an arm could be lifted for its successful overthrow, upon legal grounds, but according to our law as a profession—and it becomes a duty to so declare—that such an institution would be unprofessional and irregular. Yea, more, it would, professionally, be a cheat and a swindle. Its great injury could never be told—its blighting influence would be felt for ages. Should political hacks, pregnant with false philanthropy, travail into birth such a medical monstrosity, and should they "rig up" a chair to be dubbed "regular" in such an institution, and should such a chair be tendered to any regular physician, would he spurn or accept it? If he should abide the dicta of medical law, he could not accept it—no consideration could induce him to do so. If, however, he should accept it, and join hand in hand with the apostles of medical isms, and still claim to be under the law, and therefore regular—what would be the verdict of the profession? They would be compelled to declare him irregular. Upon what ground? Because of affiliation. By affiliating with men declared to be irregular, he would virtually endorse such irregularity, and in endorsing irregularity, he would, to the extent of his influence, sustain the dogmas of the heterodox, and to the same extent deny the faith, and set at defiance the law which demands obedience as a duty at his hands, if he would reap the privileges and benefits it confers.

Suppose this misguided brother, by skillful management, should so influence the Board of Trustees of such an institution as to cause them to fill its chairs with regular physicians, *at the time*, in harmony with the profession and obedient to the law, what then would be the status of the institution and its faculty? It would then not only be irregular, but illegal—both professional and statutory law would be violated, and the last state of such an institution would be worse than the first. Why? The institution could not be represented in the National Medical Association. It would be illegal, because its charter had been perverted, and irregular because of the unprofessional privileges granted by the charter.

Suppose, in order to avoid the difficulties of the case, the charter should be amended so as to make the institution regular, as to the character of its professorship, but should place it "under the patronage of the government," "instruction to be free, and accessible to all, irrespective of sex" and color, would it be regular? Would it be admitted to representation in the National Association? Certainly not. Why? Because that Association has not as yet, admitted as members, either females or negroes. The profession has too exalted an estimation and appreciation of the character of woman to degrade her to the disgusting scenes and humiliating spectacles of professional life. They do not desire to disrobe her of the purity, the tenderness, the holy influence she wields at home and in society, by bringing her in contact with vice, and arming her with the knife and saw of the Surgeon. They would not degrade her. Women, as a mass, do not desire it; there is enough of good material among the sterner sex yet left, out of which to make doctors and surgeons. The profession does not desire to degrade itself by admitting negroes within their ranks upon terms of professional equality. Socially they are, and never can be, upon terms of equality—they never can become eligible to the white race, in marital relations; they never can, socially nor professionally.

Should the National University ever become established, it could not be recognized by the profession or be admitted to representation in the Association, unless its charter should be in harmony with the constitution and code of ethics of that body, and be able to present the same credentials required of other institutions—to wit, that it is in good standing. We desire ardently to see a practical, harmonious solution of the question of medical education. All admit the faulty construction, and the unharmonious elements of the present system. While there is much to commend, there is still more to disapprove. We claim for every regular school perfect equality—equal standing and equal privileges. The profession has the right to determine the standing, and to declare what its requirements shall be towards its recognized schools. The difficulty in harmonizing the profession upon medical education lies not so much in the profession itself, as in the jealousies and the disgraceful struggles on the part of institutions, for patronage. When Colleges and their Board of Trustees shall agree to be controlled by the profession at large, as indicated through its organized associations, then a solution of all difficulties upon the subject will be had. Should they refuse to be so controlled, then the profession must and will assert its right and power to bring them to terms or pronounce them unworthy of its support and patronage.

Should, however, this National University comply with the requirements of the profession, we apprehend the profession would, sooner or later, be compelled, upon the principles of justice and ethics, to withhold its endorsement and support. Why? Because the profession can never ally itself to political partyism, or permit itself controlled by political influences. Under Government patronage, in a country like ours, where party spirit runs high, and parties are ever changing, such an institution would most likely be a puppet in the hands of political harlequins. Individual and professional rights would doubtless be ignored and trampled upon to the injury of the good, and for the promotion of the bad. To illustrate: Suppose the Board of Trustees of such an institution should, under the influences above alluded to, place in position men unworthy alike of social and professional esteem—men not recognized by us as organizations—though the charter might be unobjectionable—would the profession recognize it? Could it do so, and maintain its principles? Certainly not.

It will be remembered by the old members of the American Medical Association that some years ago one of its gifted members committed a breach of the ethics which resulted in his expulsion. Another member—a personal friend—a highly educated and worthy gentleman—had endorsed the medical qualifications of the first, after his expulsion, for which endorsement he was arraigned before that body. This gentleman apologized for committing an unprofessional act—this was not sufficient. The Association required that he should not only apologize for having endorsed the medical qualifications of the excluded member, but that he should express *his regret* that he had done so. There was no alternative; he was required either to confess his regrets for his endorsement, or on the other hand, by refusing to do so, to suffer the penalty of expulsion. He did express his "*regret*" and the Association was satisfied; nothing short of this would have satisfied it. Had he refused to have complied with this requisition, and had been expelled, what would have been his position professionally? Would he not have been placed upon the same footing with the gentleman he had originally endorsed? If his "regrets" saved him from expulsion, could he after this have affiliated with the expelled member and maintained his professional integrity? If he had been expelled, could he have claimed all the rights, privileges and benefits of the profession? Could his personal friends have affiliated with him, being expelled, without placing themselves in the same position he had assumed towards the friend he had endorsed, and in consequence of which he was forced to abandon or be himself expelled?

If the Association could not permit one of its individual members to endorse even the qualifications of an expelled member, how could it, with any consistency, permit a portion of a faculty to remain in connection and affiliation with parties who had been previously expelled? Would it not be necessary for these to withdraw from the institution—or refusing to do so, would it not be the duty of the Association to expel them? When this is done, could the profession, anywhere, recognize them or the institution, its board of trustees, its students or graduates? Could the profession give such a faculty and college its patronage and support? No college can be sustained without the support and patronage of the profession. If it could not receive patronage from the regular profession, it certainly could not draw its support from the ranks of eclectics, hydropathics and homœpathics, without a change of its charter. To do so, would make it both illegal and irregular.

But suppose such an University—operating under a Board of Trustees, appointed at the suggestion of political partiality, to carry out private ends, and to subserve individual interest—should expel one of the faculty to make room for some Presidential or Congressional friend? Suppose this expelled member of the faculty was universally esteemed for his eminent talent and qualifications as an instructor, what would be the sentiment of the profession in view of such an outrage upon individual rights and professional interests? Could there be found, anywhere, an individual who would consent to accept a position vacated by such means? If any individual should consent to do so, would he not, in accepting it, subject himself to the condemnation of the profession and render himself obnoxious to the principles and penalties of the code of ethics? Yea—worse, would he not subject himself to the frowns of all honest men, of every class?

The principles upon which the profession is based are so just and honorable, that each individual member is in duty bound not only to conform to the mandates of ethical law, but that he shall place himself firmly and resolutely against all those who violate the same.

We are not opposed to the establishment, by Congress, of a National University, if at the same time it be placed under the control of a Board of Trustees with a charter requiring them to conform strictly to those principles which now govern regular schools, and dissevered entirely from the intrigues and manipulations of politicians. We confess we do not hope for this, under the scheme proposed, nor indeed, under any other scheme; and, therefore, we would be recreant to the profession and the cause of medical education, should we suffer our pen to endorse a proposition so full of hazard to the best interests of science and an honored profession.

---

